# A hidden reservoir of integrative elements is the major source of recently acquired foreign genes and ORFans in archaeal and bacterial genomes

**DOI:** 10.1186/gb-2009-10-6-r65

**Published:** 2009-06-16

**Authors:** Diego Cortez, Patrick Forterre, Simonetta Gribaldo

**Affiliations:** 1 Institut Pasteur, Département de Microbiologie, Unité de Biologie Moléculaire du Gène chez les Extrêmophiles, Rue du Dr Roux, 75 724 PARIS cedex 15, France

## Abstract

**Background:**

Archaeal and bacterial genomes contain a number of genes of foreign origin that arose from recent horizontal gene transfer, but the role of integrative elements (IEs), such as viruses, plasmids, and transposable elements, in this process has not been extensively quantified. Moreover, it is not known whether IEs play an important role in the origin of ORFans (open reading frames without matches in current sequence databases), whose proportion remains stable despite the growing number of complete sequenced genomes.

**Results:**

We have performed a large-scale survey of potential recently acquired IEs in 119 archaeal and bacterial genomes. We developed an accurate *in silico *Markov model-based strategy to identify clusters of genes that show atypical sequence composition (clusters of atypical genes or CAGs) and are thus likely to be recently integrated foreign elements, including IEs. Our method identified a high number of new CAGs. Probabilistic analysis of gene content indicates that 56% of these new CAGs are likely IEs, whereas only 7% likely originated via horizontal gene transfer from distant cellular sources. Thirty-four percent of CAGs remain unassigned, what may reflect a still poor sampling of IEs associated with bacterial and archaeal diversity. Moreover, our study contributes to the issue of the origin of ORFans, because 39% of these are found inside CAGs, many of which likely represent recently acquired IEs.

**Conclusions:**

Our results strongly indicate that archaeal and bacterial genomes contain an impressive proportion of recently acquired foreign genes (including ORFans) coming from a still largely unexplored reservoir of IEs.

## Abstract 2

A large-scale survey of potential recently acquired integrative elements in 119 archaeal and bacterial genomes reveals that many recently acquired genes have originated from integrative elements

## Background

Integrative elements (IEs) such as viruses and plasmids and their associated hitchhiking elements, transposons, integrons, and so on, mediate the movement of DNA within genomes and between genomes, and play a key role in the emergence of infectious diseases, antibiotic resistance, biotransformation of xenobiotics, and so on [1-3]. Traces of IE activity have been highlighted in many prokaryotic genomes, which carry different repertoires of inserted prophages, plasmids, transposons and/or genomic islands [4-7]. These few characterized IEs are most likely only a reflection of a more diverse and still unknown IE universe that shapes bacterial and archaeal genomes [8].

The importance of IEs in the origin of ORFans (open reading frames (ORFs) without matches in current sequence databases) [9] is still controversial. Indeed, the source of ORFans remains a major mystery of the post-genomic era since, contrary to previous expectations, their proportion remains stable despite the increasing number of complete genome sequences available [10]. It has been suggested that ORFans are either misannotated genes, rapidly evolving sequences, newly formed genes, or genes recently transferred from not yet sequenced cellular or viral genomes [10,11]. The possibility that ORFans originate from the integration of elements of viral origin is appealing since viral genomes themselves always contain a high proportion of ORFans [12,13]. Consistent with this hypothesis, Daubin and Ochman [14] noticed that ORFans from γ-Proteobacteria share several features with viral ORFans (for example, small size, AT-rich) and suggested that 'ORFans in the genomes of free-living microorganisms apparently derive from bacteriophages and occasionally become established by assuming roles in key cellular functions.' However, Yin and Fisher [10] recently reported that, on average, only 2.8% of all cellular ORFans have homologues in current viral sequence databases, raising doubts about the hypothesis of a viral origin of ORFans, and proposed that 'lateral transfer from viruses alone is unlikely to explain the origin of the majority of ORFans in the majority of prokaryotes and consequently, other, not necessarily exclusive, mechanisms are likely to better explain the origin of the increasing number of ORFans.' More recently, the same authors found that only 18% of viral ORFans (ORFs present in only one viral genome) have homologues in archaeal or bacterial genomes, and concluded that 'phage ORFans play a lesser role in horizontal gene transfer to prokaryotes' [12].

Several *in silico *methods based on composition have been conceived in the past few years to identify foreign genes that were recently acquired by cellular genomes, such as atypical G+C content, atypical codon usage, Markov model (MM)-based approaches, and Bayesian model (BM)-based approaches [5,6,15-22]. MM approaches are based on one-order Markov chains to identify those ORFs that have a composition different from genes that are likely native [15], whereas BM approaches identify those ORFs with under-represented compositions with respect to the composition of the whole genome (see [16] for details). Composition-based methods are based on the idea that foreign DNA fragments acquired either from distant cellular sources or from IEs can be identified by the fact that they harbor atypical sequence signatures with respect to the host genome. Indeed, genomic signatures differ between distantly related organisms [23] and it has been shown that viruses and plasmids might keep a distinct dinucleotide signature with respect to that of their hosts [24-26]. The accuracy of most of the compositional methods designed to detect horizontally transferred genes has not been validated statistically. Here, we have sought to quantify more precisely the role of IEs in the introduction of foreign genes in bacterial and archaeal genomes and in the origin of ORFans. We have developed an accurate and statistically validated MM-based strategy to search 119 archaeal and bacterial genomes for 'clusters of atypical genes' (CAGs), since these likely represent recently integrated foreign elements, including IEs.

## Results and discussion

### Identification of atypical ORFs

**Figure 1:**
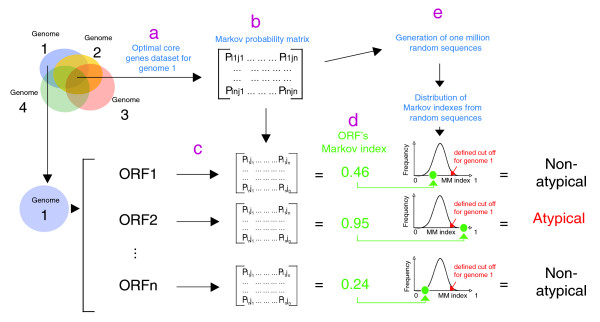
Markov model-based strategy. **(a) **An optimal core genes dataset is determined, and **(b) **a Markov probability matrix is built. **(c) **For a given genome, each ORF is analyzed using a Markov model that takes into account the Markov probability matrix of the core gene dataset and the composition of the ORF under study. **(d) **Fore each ORF the model calculates an index that represents the likelihood of that ORF having a composition similar to the core genes dataset. **(e) **One million random sequences are generated based on the Markov probability matrix of the core genes dataset, and their Markov indexes are calculated. **(f) **ORFs having a Markov index below a defined threshold of the distribution of random sequence indexes are considered as atypical.

Recently, using *in silico *horizontal gene transfer (HGT) simulations in the *Escherichia coli *K12 genome, we tested the performances of different composition-based methods: atypical G+C composition, atypical codon usage, MM approaches, and BM approaches [15]. Whereas the first two methods displayed rather low performance, the MM and BM approaches were able to detect artificially introduced foreign genes quite accurately [15]. Here, we have extended the MM approach by taking advantage of the availability of a large number of genomes from closely related organisms. The general strategy is the following (Figure 1): for different groups of closely related genomes, a dataset of conserved orthologues (hereafter referred to as 'core genes') is extracted (Figure 1a); this is used to build for each genome a refined Markov probability matrix that represents its genomic composition signature (Figure 1b); then, for a given genome, the MM model analyzes each ORF by taking into account the Markov probability matrix of the core genes dataset and the composition of the ORF under study (Figure 1c); the MM model calculates for each ORF an index that represents the likelihood of that ORF to have a composition similar to the core genes dataset (Figure 1d); for each ORF, one million random sequences are generated based on the Markov probability matrix of the core gene dataset, and their Markov indexes are calculated (Figure 1e); finally, ORFs having a Markov index above a defined cut-off are considered as atypical (Figure 1f).

**Figure 2:**
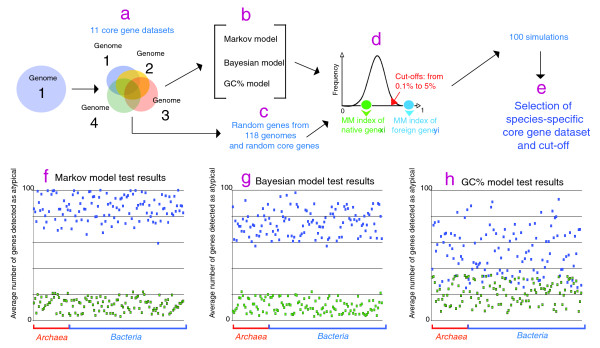
HGT simulations. **(a) **Eleven core gene datasets for each analyzed genome were determined and, for each genome, 11 Markov models were built based on these different gene datasets. **(b) **The efficiency of our MM approach, the BM approach, and a GC% approach to detect foreign ORFs was tested by performing *in silico *HGT simulations using a variety of core gene datasets. **(c) **For the HTG simulations, 100 genes were chosen from the other 118 genomes and 100 random core ORFs were *in silico *introduced in the genome under analysis. **(d) **The average number of these ORFs that were detected as atypical (false positives, expected to be low) was determined. **(e) **After 100 simulations we searched for the core genes dataset and the cut-off where the average detection of simulated HGT was the highest but the average detection of native core genes was the lowest. **(f-h) **Average result after 100 HGT simulations for the 119 analyzed genomes using the MM, BM and GC% methods with species-specific core gene datasets and cut-offs. Blue dots represent the average number of true positives detected. Green dots represent the average number of false positives detected. The MM method had a significantly higher rate of detection of true positives than the BM method (Wilcoxon test W = 11,849, *P*-value < 2.2 e-16, means = 86.8 and 74.8 for the MM and BM methods, respectively; and Wilcoxon-test W = 13,824, *P*-value < 2.2 e-16, means = 86.8 and 52.6 for MM and GC%, methods, respectively). No significant differences were found between the MM and BM methods in the detection of false positives (Wilcoxon-test W = 8,359, *P*-value = 0.0311, means = 12.4 and 11.0 for the MM and BM methods, respectively).

We selected 19 groups of closely related archaeal and bacterial genomes (for example, same genus or order, 119 genomes in total) representing a good sampling of prokaryotic diversity. To define 'core genes' datasets, best bi-directional BLASTP searches were performed with the ORFs of each genome against those of the other members of the group. All hits having a bit score higher than 30% of the bit score of the seed against itself were considered as orthologues [27]. The core genes datasets are essential for the MM to work properly. In fact, its ability to detect atypical ORFs depends entirely on the probability matrix of the model. For instance, if very few genes are included in the core genes dataset, the matrix will be small and this will increase the number of detected atypical genes artificially. On the other hand, a larger probability matrix (an extreme case being a matrix built with all the genes of a genome) would reduce dramatically the model's detection ability for atypical ORFs. Thus, it is essential to define for each genome an optimal dataset of core genes to obtain the best performance of the MM model. For each analyzed genome, we created 11 datasets of core genes: all the genes in the genome, orthologues present in 10% of the group's genomes, orthologues present in 20% of the group's genomes, and so on, up to orthologues present in 100% of the group's genomes. Then, for each genome, we built 11 MMs based on these different core genes datasets (Figure 2a). We tested the efficiency of our MM approach, the BM approach, and a GC% approach to detect atypical ORFs by performing *in silico *HGT simulations in all 119 archaeal and bacterial genomes (Figure 2b; Materials and methods). We performed two types of HGT simulations using all 11 different core genes datasets. In the first simulation we chose 100 ORFs from the other 118 genomes (Figure 2c) and these were introduced *in silico *in the genome under analysis. We then determined the number of simulated HGTs that were detected as atypical (true positives, expected to be high). In the second simulation, we chose 100 random ORFs from a strict core genes dataset (that is, genes conserved in all genomes of the group, thus assumed to be native; Figure 2c) and we determined the average number of these that were detected as atypical (false positives, expected to be low; Figure 2d; see Materials and methods). For both simulations, the results were analyzed by a one-tailed test with different distribution cut-offs (0.l% to 5%). Then, we identified the core genes dataset and the cut-off for which the average detection of simulated HGT (true positives) was the highest but the average detection of native core genes (false positives) was the lowest (Figure 2e). These parameters allowed the definition of an optimal core genes dataset and cut-off for each genome analyzed (Table S1 in Additional data file 1), which is thus independent of the evolutionary scale of each group of genomes analyzed. The HGT simulations were carried out with the same random ORFs for the three models (MM, BM and GC% approaches). Although there were no significant differences between the MM and BM methods in the number of false positives, the MM method had a statistically significantly higher rate of detection of true positives than the BM and the GC models (Figure 2f-h).

### Identification of CAGs

We thus applied the MM method to detect ORFs with atypical composition in the 19 groups of closely related archaeal and bacterial genomes, using the optimal genome-specific core genes datasets and cut-offs. This led to the identification of 58,487 ORFs of atypical composition in the 119 genomes (Table 1; Table S1 in Additional data file 1). As a control, a high fraction (85%) of the ORFs that localized within 275 already annotated integrated elements were detected as atypical by our method, while only 56% of them were detected as atypical by the BM approach and 36% by the GC% approach (data not shown). This confirms that recently integrated foreign elements harbor a sequence signature distinct from that of their hosts that can be detected with appropriate methodologies.

**Table 1:** General information of analyzed genomes, newly identified CAGs and ORFans

**Analyzed genomes**	
Number of genomes	119
Number of ORFs	351,111
Number of atypical ORFs	58,487 (16% of all genes)
Number of CAGs	2,377
Number of ORFs in CAGs	47,441 (13% of all genes)
	
**Newly identified CAGs**	
Number of CAGs	2,104
CAGs of likely plasmid origin	674 (32%)
CAGs of likely virus origin	164 (8%)
CAGs of likely viruse and plasmid origin	341 (16%)
CAGs likely from cellular sources	145 (7%)
CAGs unclassified	780 (37%)
	
**ORFans**	
Number of ORFans	8,987
Number of ORFans in CAGs	3,475 (39%)
ORFans in annotated proviruses and CAGs of likely virus origin	875 (25.1% of all ORFans inside CAGs)
Number of ORFans in CAGs of likely plasmid origin	680 (19.5% of all ORFans inside CAGs)
Number of ORFans in CAGs of likely virus and plasmid origin	224 (6.4% of all ORFans inside CAGs)
Number of ORFans in CAGs likely from cellular sources	54 (1.5% of all ORFans inside CAGs)
Number of ORFans in unclassified CAGs	1,642 (47.5% of all ORFans inside CAGs)

We then searched for atypical genes that cluster together, since these may be recently integrated foreign elements. We used a sliding window of ten ORFs that moved along the genome sequence [16,18], and every time seven or more ORFs in that window showed an atypical composition we defined a cluster (that may thus also include non-atypical genes). This threshold of seven genes was based on the distribution of atypical versus non-atypical genes observed in annotated IEs. We applied this protocol to each of the 119 genomes and identified a total of 2,377 CAGs (Table 1; Table S1 in Additional data file 1). The CAGs include as high as 13% of all ORFs analyzed (Table 1; Table S1 in Additional data file 1), indicating that the integration of foreign elements into archaeal and bacterial genomes is very frequent. We verified whether our method has a tendency to identify clusters of small atypical ORFs; however, ORFs included in CAGs have statistically the same size distribution as core genes (Figure S1 in Additional data file 2).

**Figure 3:**
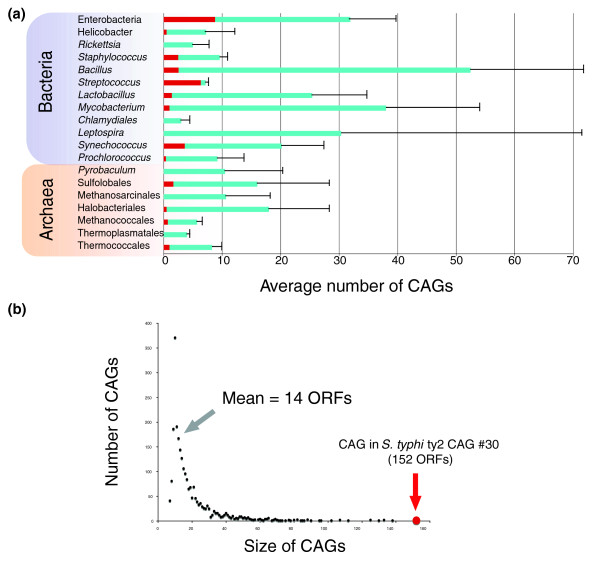
Number of identified CAGs, CAG size distribution and proportion of already annotated IEs. **(a) **Average number and standard deviations of CAGs in the different analyzed groups of Archaea and Bacteria. In red are represented, for each group, the average numbers of annotated IEs. **(b) **CAGs size distribution.

The number of CAGs varied greatly among and within groups of genomes (Figure 3a), being, on average, between 10 and 30, with a minimal number for *Rickettsia *and *Chlamydia *(that is, zero in *Rickettsia typhi*, *Rickettsia prowazekii*, *Chlamydia abortus *and *Chlamydia felis*) (Table S1 in Additional data file 1). The size of CAGs (expressed as the number of ORFs included) varied from seven (by definition) to several hundreds (up to a 152 ORF CAG in the γ-proteobacterium *Salmonella typhi *ty2; Figure 3b). Interestingly, archaeal genomes harbor, on average, half as many CAGs as Bacteria (Additional data file 1), suggesting that Archaea are somehow less prone than Bacteria to the integration of foreign elements. Nevertheless, some archaeal genomes exhibit a high number of CAGs, such as *Haloquadratum walsbyi *(32 CAGs) and *Sulfolobus solfataricus *(28 CAGs). In these two cases, the great majority of CAGs are concentrated within specific regions of the chromosome, near potential replication termination areas, at 150° from OriC in H. walsbyi and between the second and the third origin of replication in S. solfataricus (two regions in *H. walsbyi *and one in *S. solfataricus*; Additional data file 3). Moreover, small clusters of native genes (from 5 to 30 ORFs) separate these CAGs, suggesting that chromosomal rearrangements may have fragmented a larger integrated original element (Additional data file 3). Some bacterial groups also exhibited a very high number of CAGs. *Mycobacterium*, *Lactobacillus*, *Bacillus *and enterobacteria genomes are good examples, because they all contain between 30 and 100 CAGs (Additional data file 1).

As mentioned for *H. walsby*, we noticed an enrichment of CAGs in particular chromosomal regions, which are separated by small clusters of native genes (Additional data file 3). Furthermore, the frequency of CAGs in archaeal and bacterial genomes is inversely proportional to their size, indicating that larger CAGs are less frequent than smaller ones (Figure 3a). This suggests again that CAGs are quickly fragmented and/or eroded following integration. Moreover, CAGs are enriched in pseudogenes, since they include 32% of all pseudogenes (of those annotated in bacterial genomes together with those recently identified in a large number of archaeal genomes [28]) (data not shown).

### Origin of CAGs

The number of CAGs corresponding to already annotated IEs is larger in well-annotated genomes such as those from enterobacteria and *Streptococcus *(Figure 3a, red), while for the majority of other genomes the number of newly identified CAGs is high (Figure 3a, blue).

tRNA and tmRNA genes are well known integration points for IEs [29,30]. In archaeal and bacterial genomes, around 40% of already annotated IEs are found next to tRNA and tmRNA genes (Additional data file 3). Moreover, a significant proportion of all newly identified CAGs (33.27%) also lies next to a tRNA or tmRNA gene (Table S1 in Additional data file 1), strongly indicating that many of these may be recently acquired IEs. In some groups, the number of newly identified CAGs representing potential recently acquired IEs appears particularly important. For instance, in enterobacteria, *Streptococcus*, *Prochlorococcus *and Thermoplasmatales, between 40% and 50% of their CAGs lie next to tRNA genes. On the contrary, all groups of closely related Firmicutes genomes show a lower number of newly identified CAGs lying next to tRNA genes (less than 5%; Additional data file 1). Interestingly, in the genomes from Firmicutes, tRNA genes are grouped in a reduced number of large clusters (Additional data file 3), suggesting that such clustering may somehow make the integration of IEs more difficult. *Lactobacillus *genomes are an exception within Firmicutes, because CAGs often lie next to a tRNA gene (which are also less clustered; Additional data file 3) - for example, in *Lactobacillus gasseri *15 of 16 CAGs are found next to tRNA genes (Additional data file 1).

**Figure 4:**
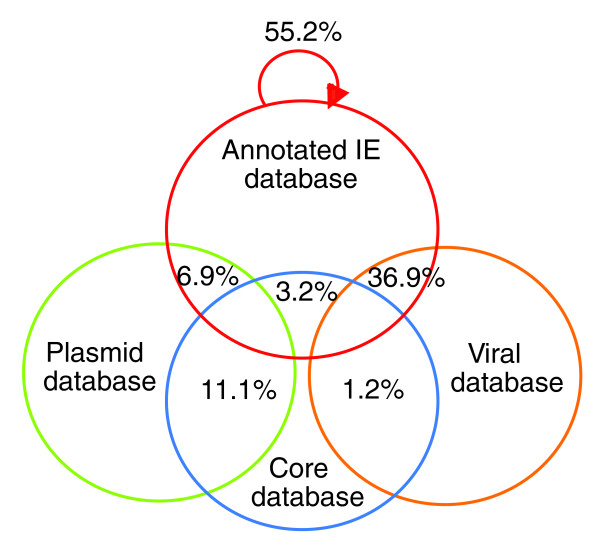
Proportion of homologues from annotated IEs, newly identified CAGs, and core genes in various databases. Proportion of homologues of ORFs from annotated IEs in the core genes database, the viral database, the plasmid database and the annotated IE database, as well as the proportion of homologues of core genes in the viral database and the plasmid database.

In summary, around 30% of newly identified CAGs lie next to tRNA genes and are thus likely to have been derived from IEs. However, the remaining CAGs may also be IEs because not all IEs integrate next to a tRNA or a tmRNA gene, or because they may have been displaced or fragmented following genomic rearrangements. We thus sought to obtain more information on the source of our newly identified CAGs by analyzing their gene content. For this, we developed a probabilistic approach that would help us determine if a given CAG is of IE origin by looking at its gene content. This approach is based on the calculation of the probability to have a certain number of homologues in a database of known IE sequences. Briefly, it calculates, using Monte Carlo simulations, the most probable source at 95% confidence intervals. Nevertheless, for this approach to work, IE sequences must represent a separate gene pool from cellular sequences. Therefore, we first compared a local database containing all ORFs from annotated IEs (annotated IE database) with a local database containing all our species-specific core genes from all genomes analyzed (core database), with the complete viral genome database available at NCBI (as for January 2009; viral database) (Figure 4a, b), and with the complete plasmid genome database at NCBI (as for January 2009; plasmid database) (see Materials and methods). As expected, annotated IEs share a large number of homologues with the NCBI viral database (36.9%; Figure 4) and with other annotated IEs (55.2%; Figure 4), generally from closely related genomes (data not shown), indicating the existence of evolutionarily related IEs. Interestingly, the annotated IE database has in common only a minor fraction of homologues with the core database (3.2%; Figure 4) and with the plasmid database (6.9%; Figure 4). Consistently, core genes share a rather low number of homologues with the viral database (1.2%; Figure 4) and with the plasmid database (11.1%; Figure 4). These results clearly indicate that the pools of viral genes and plasmid genes are separated from the pool of core genes.

**Figure 5:**
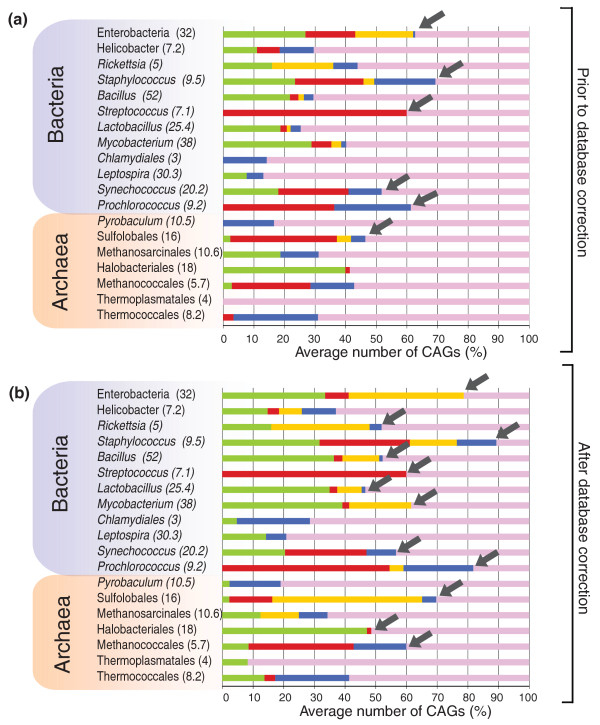
CAGs of likely IE origin based on probabilistic analysis. **(a) **Proportion of newly identified CAGs of plasmid origin (green) for each analyzed group; proportion of identified CAGs of viral origin (red); proportion of newly identified CAGs of viral/plasmid origin (yellow); proportion of newly identified CAGs of cellular origin (blue); proportion of newly identified CAGs that are unassigned (violet). **(b) **Same as in (a) but after database correction. Each group's average number of CAGs is indicated in parentheses. Grey arrows indicate the groups with the highest proportions of newly identified CAGs classified as IEs.

Given this clear distinction, we could apply our probabilistic approach to distinguish the newly identified CAGs that are related to known viruses and plasmids (see Materials and methods). Since annotated IEs are clearly related to viruses, we incorporated them into the viral database. We calculated 95% confidence intervals able to assign a CAG as related to viral or plasmid sources. To do so, we artificially constructed 1,000 clusters containing different numbers of ORFs (7 > *n *< 152) and analyzed the presence of homologues in the viral and plasmid databases. Next, for each *n *we built a distribution of probabilities and defined 95% confidence intervals. CAGs were then assigned a given origin (viral or plasmid) if the number of homologues was above the 95% confidence interval corresponding to their size (see Materials and methods for details). As expected, 95% of annotated IEs were correctly assigned as of viral origin (data not shown). For the newly identified CAGs, 21% were assigned as of plasmid origin (Figure 5a, green), 8% as of viral origin (Figure 5a, red) and 4% as of plasmid/viral origin (CAGs with equal probabilities of being of plasmid and viral origin; Figure 5a, yellow). Nevertheless, the origin of an important proportion of newly identified CAGs (67%) could not be assigned to either viral or plasmid sources by this approach. This may be due to the fact that these CAGs are related to viral or plasmid sources that are not yet sequenced and thus not included in the viral and plasmid databases. In order to reduce this bias, we added to their corresponding databases the ORFs from all newly identified CAGs that were assigned to viral or plasmid origin, and we performed a second round of analysis. All CAGs assigned to viral or plasmid origins were then added to their corresponding databases, and the analysis was repeated. At the fifth iteration, no more CAGs could be assigned. After this new analysis, aimed at correcting a bias in the original databases, the percentage of newly identified CAGs assigned to plasmid origin was raised to an average of 32% (Figure 5b, green; Table 1), while the percentage of CAGs assigned to viral origin remained almost unchanged (8% on average; Figure 5b, red and blue; Table 1). Interestingly, the number of CAGs of plasmid/viral origin increased to an average of 16% (Figure 5b, yellow; Table 1; see, for example, enterobacteria and Sulfolobales). These results indicate that the majority of new CAGs correspond to large IE families of both plasmid and viral origin.

After this database correction, not only just a few specific groups, but many archaeal and bacterial genome groups were shown to harbor high proportions of newly identified CAGs assigned to either plasmid, viral, or plasmid/viral origins (Figure 5b, grey arrows). For instance, we were able to determine that *H. walsbyi *harbors a large fragmented plasmid in a particular genomic region (Additional data file 3) since the majority of CAGs in this region are statistically related to plasmids (Additional data file 3). *S. solfataricus *also contains a large fragmented element, which is equally related to viruses and plasmids (Additional data file 3). In fact, many other CAGs in the genomes of Sulfolobales are also related to both viral and plasmid sequences (Figure 5b; Additional data file 3). A similar case is observed in the genomes of enterobacteria (Figure 5b; Additional data file 3).

It is interesting to note that the major source of recently acquired IEs can change from group to group. For instance, in all genomes of Firmicutes and Halobacteriales, the great majority of CAGs are related to plasmids, whereas in *Procholococcus*, *Streptococcus *and Methanococcales, they are rather related to viruses (Figure 5b; Additional data file 3). The analyses of *Leptospira interrogans *and *Mycobacterium leprae *genomes nicely reflect the different histories that can lead to a high number of CAGs in a given genome. *L. interrogans *harbors 80 CAGs including more than 1,400 genes (Additional data file 1). On the contrary, *M. leprae *harbors 58 CAGs including 1,500 genes, but over 40% of its ORFs are annotated as pseudogenes. Here, CAGs do not represent IEs being erased but rather reflect the important genomic reduction that is ongoing in this species (Figure 5b; Additional data files 1 and 3). To summarize, 56% are likely recently acquired IEs.

To test the origin of the remaining CAGs, we repeated the same probabilistic analysis against the cellular database. Only 4% (7% after database correction) could be assigned as being of cellular origin, meaning that these may be either elements recently acquired by HGT from cellular sources, or else clusters of atypical native genes (Figure 5b; Additional data files 1 and 3).

The origin of the remaining 37% of newly identified CAGs remained unassigned even after database correction (Figure 5b, violet; Table 1). Given the current under-representation of viral/plasmid diversity in public sequence databases rather than cellular diversity, these elements are probably members of new families of IEs that have no sufficient viral/plasmid relatives in current sequence databases to be unambiguously assigned based on our probabilistic approach. Importantly, only 5.96% of newly identified CAGs have homologues (<e^-5^) in four local viral metagenomic databases (see Material and methods), but these databases may be also be biased towards particular viral lineages.

### ORFan distribution in CAGs

We identified 8,428 ORFans in the 119 complete genomes (defined as ORFs presenting no Blast hits against the nr database at the NCBI below an e-value of 0.001 [9]; see Materials and methods; Table 1; Table S1 in Additional data file 1). These ORFans are thus likely to be genes of very recent origin. Nearly all of them (96%) have atypical sequence composition and they are, on average, statistically smaller than non-ORFans (Figure S2 in Additional data file 2). Interestingly, this is also valid for ORFans from complete sequenced viral genomes (Figure S2 in Additional data file 2).

**Figure 6:**
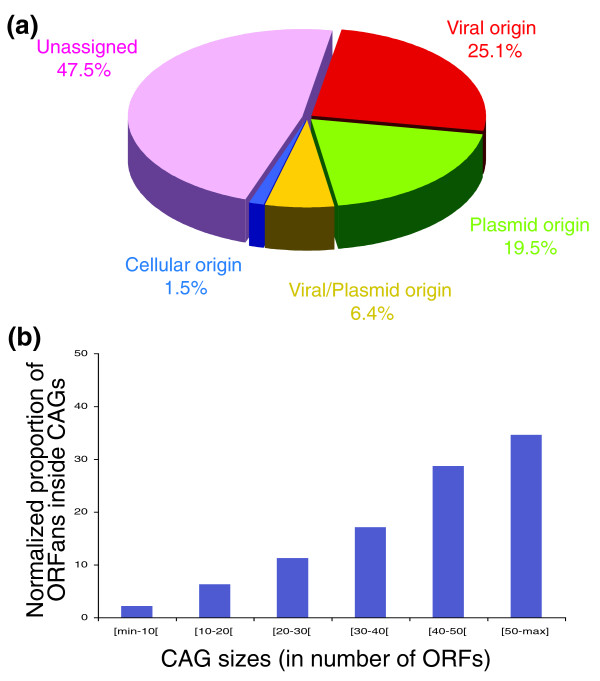
ORFan distribution. **(a) **Distribution of ORFans in CAGs: ORFans in CAGs of viral origin (red); ORFans in CAGs of plasmid origin (green); ORFans in CAGs of viral/plasmid origin (yellow); ORFans in CAGs of cellular origin (blue); and ORFans in unassigned CAGs (violet). **(b) **Proportion of ORFans inside CAGs of different sizes. Data were normalized according to the number of CAGs in each category.

We analyzed the distribution of these ORFans. We observe that 39% of all ORFans lie inside CAGs (Table 1), and a χ^2 ^statistical test shows that this is more frequent than would be expected by chance only (Figure S3 in Additional data file 2). Importantly, genomes with a high number of ORFans (more than 200; Additional data file 1), such as those of *L. interrogans*, *Methanosarcina acetivorans*, *H. walsbyi*, and *Lactobacillus casei*, have around 50% of their ORFans inside CAGs. Moreover, not only small ORFans, but also ORFans with more than 100, 150 and 200 amino acids (thus likely to be genuine coding genes rather than misannotated genes), are overrepresented in CAGs (37.8%, 37.3% and 37.4%, respectively; data not shown). Interestingly, even if CAGs inferred to be of plasmid origin are more numerous, ORFans are more abundant in CAGs inferred to be of viral origin (25.1%; Figure 6a, red) than in CAGs inferred to be of plasmid origin (19.5%; Figure 6a, green). Moreover, 6.4% of ORFans lie in CAGs inferred to be of plasmid/viral origin (Figure 6a, yellow), and only 1.5% of ORFans lie inside CAGs inferred to be of cellular origin (Figure 6a, blue). However, a significant proportion of ORFans (47.5%) lie inside unclassified CAGs (Figure 6a, violet).

Finally, large CAGs (that is, containing 40 or more ORFs) harbor proportionally more ORFans than smaller CAGs (Figure 6b). Since larger CAGs may be more recent than smaller ones (these likely deriving from disruption of larger CAGs), this suggests that recently acquired CAGs contain proportionally more ORFans, and that these are rapidly removed from cellular genomes. The remaining 61% of ORFans do not lie in any CAG. However, if we relax the criterion for CAG definition (from four up to six atypical genes), nearly a third of them lie in small CAGs, which may represent the remnants of older IEs (data not shown).

## Conclusions

It is widely assumed that most cellular genomes harbor IEs, but their proportion has not been quantified on a large scale. With this study, we show that the use of a MM-based method to identify ORFs with atypical composition in groups of closely related genomes, coupled to the identification of CAGs, their genomic context and gene content, is a powerful approach to identify foreign elements that have recently integrated into archaeal and bacterial genomes. This strategy allowed us to recognize all previously annotated IEs and to detect new CAGs that are likely of viral or plasmid origin in a large number of archaeal and bacterial genomes.

The MM approach that we have developed could have many useful applications. In particular, it may help automatic genome annotation (detection of IEs in newly sequenced genomes) and will allow an exhaustive description of all IE families in sequenced archaeal and bacterial genomes. Our MM method could also be very useful to determine if an ORF of interest belongs to an IE. For instance, we have recently used it to show that a conserved ORF (AFV3) from viruses infecting Crenarchaeota has a unique homologue outside this viral group that lies inside a CAG corresponding to a conjugative transposon in the genome of *Bacillus subtilis *[31,32]. Interestingly, this transposon is related to a large family of CAGs that includes many of our newly identified CAGs, as well as annotated prophages, 'free-living' phages and plasmids from Firmicutes [31]. This result highlights unsuspected and still largely unknown links between the archaeal and bacterial IE worlds.

Our results are also different from those recently reported by Hsiao and colleagues [6], who used a method based on atypical dinucleotide composition to identify genomic islands in 63 prokaryotic genomes. However, in contrast to our MM strategy, they failed to identify more than half of already annotated IEs. This significant difference between the two methodologies underscores the importance of performing HGT simulations to test statistically a method's accuracy prior to its application.

Importantly, we found that several bacterial and archaeal genomes contain an impressive number of CAGs, whereas others contain only a few. These results are not correlated with gene number nor with the evolutionary proximity of the genomes in a given group. Different bacteria and archaea are thus differently prone to acquire IEs. This may be linked to different adaptation strategies under high selection pressures [33] and might be related to a general mechanism for niche differentiation in microbial species [34] by increasing the genetic variability via the acquisition of foreign genetic material.

Among our newly identified CAGs, a large number are inferred to be of plasmid origin. This indicates that plasmid integration is highly frequent, which is at odds with previous reports. For example, Nakamura and colleagues [16] concluded that most of the atypical ORFs that they identified are from distant cellular sources since only 30% of these have homologues in viral or plasmid sequence databases. We showed that these databases are, in fact, biased towards viruses and plasmids from particularly well-characterized organisms. When this bias was corrected, a larger number of CAGs could, in fact, be assigned to plasmid/viral origin. Future increases in the number of available new viral, plasmid and cellular sequences will allow the large number of CAGs that remained unclassified in our analysis to be classified and may also be of viral or plasmid origin.

Importantly, our results contribute to the issue of the origin of ORFans in archaeal and bacterial genomes. In fact, a large percentage of ORFans were found to lie in CAGs, half of which are new CAGs of likely viral or plasmid origin. The number of ORFans included in CAGs increased to up to 60% if we also considered smaller CAGs (two to six genes) that might be remnants of older/fragmented IEs.

The IE origin of most recent ORFans is consistent with the recurrent observation that viral and plasmid genomes always contain a higher proportion of ORFans than cellular genomes [13]. Our data are in agreement with those reported by Daubin and Ochman for γ-Proteobacteria [14] and indicate that the IE origin of ORFans is a phenomenon that shapes equally the genomes of both archaea and bacteria. Consequently, the reported low number of ORFans with homologues in viral databases [10], or else the reported low number of viral ORFans with homologues in bacterial or archaeal genomes [12], is very likely due to a large under-representation of viral and plasmid diversity in current sequence databases.

Our analysis strongly suggests that the variable component of a particular genome with respect to its closely related kin (that is, ORFans) has its origin in a still largely unsequenced (hidden) reservoir of IE sequences. Consistently, direct microscopic observations and metagenomic data indicate that viruses are the most abundant entities and the greatest source of gene diversity on Earth [8,35,36]. The hidden IE reservoir hypothesis also explains why the proportion of ORFans remains stable despite the growing number of new genome sequences. We predict that this proportion will start decreasing only with a more exhaustive sequencing of all IEs associated with a particular bacterial or archaeal species. The study of the expression profiles, functions and structures of these ORFans should become one of the priorities of post-genomics studies.

## Materials and methods

### Analyzed genomes

The following groups of closely related genomes were analyzed. Group I, Archaea: Thermococcales, four genomes; Methanosarcinales, three genomes; Halobacteriales, four genomes; Thermoplasmatales, three genomes; Sulfolobales, three genomes; *Methanococcus*, seven genomes; *Pyrobaculum*, four genomes. Group II, Bacteria: γ-Proteobacteria (*Escherichia*, *Salmonella *and *Yersinia*), 13 genomes; ε-Proteobacteria (*Helicobacter*), four genomes; α-Proteobacteria (*Rickettsia*), five genomes; Firmicutes (*Bacillus*, 11 genomes; *Staphylococcus*, 12 genomes; *Streptococcus*, 6 genomes; *Lactobacillus*, 10 genomes); Actinobacteria, (*Mycobacterium*), ten genomes; *Chlamydia*, 7 genomes; Spirochaetes (*Leptospira*), three genomes; *Cyanobacteria *(*Synechococcus*, five genomes; *Prochlorococcus*, five genomes). The complete list of species is given in Table S1 in Additional data file 1. All genomes were obtained from the NCBI database [37].

### Models

Eleven first-order Markov-based models were constructed for each genome for each different core genes dataset. The models take into account the Markov probability matrix of the different core genes datasets and the composition of the ORF under study. The model is based on the mathematic formulas described in [38], and summarized below:



where *S*(*m*) is the Markov index for the *m *sequence, *h *is sequence length of the gene *m*, *P*(*xy*) *set ORFi *are the dinucleotide probabilities found in the ORF *i *under study, and *P*(*xy*)*set coregeneX% *are the dinucleotide probabilities calculated from the core genes dataset calculated from gene sequences from the organisms under study having orthologues in at least X% of the group's genomes.

The model calculates for each ORF an index that represents the likelihood of that ORF to have a similar composition to the core genes dataset (that is, a Markov index close to one for a given ORF means that its composition is similar to that of the core genes dataset). In order to assess significance cut-offs for Markov indexes, we applied the following statistics (based on the method described in [16]) and Monte Carlo simulations; for every ORF of a particular group analyzed, one million random sequences were generated based on the Markov model probability matrix of the core genes dataset, and the Markov index of each of these random sequences was calculated. Then, the results were analyzed by a one-tailed test with different distribution cut-offs (0.l% to 5%). An ORF having a Markov index above a specific cut-off was then considered as atypical. The Bayesian model was built as detailed in Nakamura *et al*. [16] but with our different core genes datasets and our Monte Carlo simulations to define statistical thresholds. The GC% model looks for the differences between a give ORF and a dataset of core sequences by looking at the GC% variability in the third codon base. The model was applied using the different core genes datasets and our Monte Carlo simulations to define statistical thresholds. Genes that are atypical *per se *(approximately 10% of all core genes analyzed), such as genes coding for ribosomal proteins or genes smaller than 150 nucleotides, were excluded from further analysis.

### Horizontal gene transfer simulations

The MM, BM and GC% approaches were evaluated using *in silico *HGT simulations in order to test their performances under different genomic backgrounds. The 119 genomes were analyzed and 100 simulations were performed using the core genes datasets and a variety of cut-offs (0.1% to 5%). Higher Markov orders were also tested, but these showed lower specificity (that is, higher numbers of false positives; data not shown), probably because with our Markov chain approach the increase in the Markov order reduces considerably the quantity of information that can be obtained from the gene sequence, especially for small genes. To evaluate the average performances of the models, we applied a Wilcoxon-test.

### Homology searches

All ORFs contained in annotated IEs (10,651 ORFs) and newly identified CAGs (36,790) were searched by BLASTP [39] against: a local database of all annotated IEs in the 119 genomes; complete plasmid sequences available at the NCBI; complete viral genomes at the NCBI; a local database of core genes in the 119 genomes (from the selected core genes dataset after the HGT simulations; 194,554 genes); and ORFs in newly identified CAGs. Bit-score is useful when comparing BLAST results obtained from different databases searches because it remains constant, unlike the e-value, which changes depending on the size of the database. We therefore defined the homology cut-off between two sequences when the bit-score of the BLAST hit was above 30% of the bit-score of the query protein against itself (maximum bit-score value) [27]. We also performed BLASTP searches against four metagenomic databases available at the SDSU Center for Universal Microbial Sequencing: 'The marine viromes of four oceanic regions' [35].

### Gene content probabilistic analysis

One-thousand clusters of size *n*, where *n *goes from 7 ORFs (smaller CAG by definition) up to 152 ORFs (larger CAG found) were artificially built using ORFs from the 119 analyzed genomes. We then counted, for all clusters of *n *size, the number of homologues they have in the viral genome database, the plasmid genome database and the core genes database. ORFs were allowed to have only one homologue in each database in order to reduce any possible biases due to the presence of closely related sequences in the database that would falsely increase the number of homologues for a given ORF. Based on these data, we built three distributions of probabilities (one for each of the above-mentioned databases), and from these distributions we were able to calculate a 95% confidence interval. We could then determine which CAGs are of viral or plasmid or cellular origin by counting the number of homologues their ORFs show in the viral genome database and the plasmid database and the core genes database. For instance, a CAG of size '*x*' that has '*y*' homologues in the plasmid database could be considered of plasmid origin when '*y*' was above the 95% confidence interval calculated from the distribution of homologues in the plasmid database of the 1,000 random clusters of size '*x*'.

### Detection of ORFans

All ORFs in the 119 analyzed genomes were searched by BLASTP against the nr database at the NCBI (as of January 2009) [37]. When no hits were found below an e-value of 0.001, ORFs were considered as ORFans [9]. We corrected the list of ORFans by eliminating potential misannotated ORFs. In fact, 1,859 potential ORFans were found in more than one genome by using a BLASTN search (cut-off was fixed at 50% of bit score of the query sequence against itself). For each genome, we calculated the expected number of ORFans inside CAGs given the total number of ORFs in the genome, the total number of ORFs in CAGs, and the total number of ORFans. Because the data had a normal distribution, a χ^2 ^test was performed to determine if the number of ORFans inside CAGs was higher than expected by chance only. To analyze if CAGs are enriched in genes of small size and because data had a normal distribution, a one-way ANOVA test followed by a TukeyHSD statistical test were performed between all the groups of CAGs and 1,000 randomly chosen core genes.

### Statistics

All statistical tests were performed with the R package [40]. All other analyses were performed using in-house developed Perl scripts.

## Abbreviations

BM: Bayesian model; CAG: cluster of genes with atypical composition; HGT: horizontal gene transfer; IE: integrative element; MM: Markov model; ORF: open reading frame.

## Authors' contributions

DC carried out all analyses and simulations. DC, SG and PF conceived the study, interpreted the results and wrote the paper. All authors read and approved the final manuscript.

## Additional data files

The following additional data are available with the online version of this paper: Table S1, listing all detailed information on CAGs for the 122 analyzed genomes (Additional data file 1); supplementary Figures S1, S2 and S3 (Additional data file 2); detailed results from the 119 analyzed genomes (Additional data file 3).

## Supplementary Material

Additional data file 1 Detailed information on CAGs for the 122 analyzed genomes. Click here for file

Additional data file 2 Figure S1 shows the statistical results of the sizes of CAGs' ORFs with respect to the size of core genes. Figure S2 shows the statistics of ORF and ORFan sizes. Figure S3 shows the results of a χ^2 ^test on the frequencies of ORFans inside and outside CAGs. Click here for file

Additional data file 3 Detailed results from the 119 analyzed genomes. Click here for file
