# Supplementary material for: A hidden reservoir of integrative elements is the major source of recently acquired foreign genes and ORFans in archaeal and bacterial genomes
Source: Genome Biol. 2009 Jun 16;10(6):R65. doi: 10.1186/gb-2009-10-6-r65 (PMC2718499; doi:10.1186/gb-2009-10-6-r65)
Supplement: Additional data file 1 — Detailed information on CAGs for the 122 analyzed genomes. [file gb-2009-10-6-r65-S1.pdf]

Groups	Species	Number of genes	Number of atypical genes	% of atypical genes	Number of atypical genes in CAGs	% of atypical genes in CAGs	Number of CAGs	Number of genes in CAGs	% of genes in CAGs	Number of orphans	Number of orphans in CAGs	% of orphans in CAGs	Species-specific core gene dataset	Species-specific cut off	Number of tRNA genes per genome	Number of tRNA genes lying next to a CAG	% of tRNA genes lying next to a CAG	Number of CAGs lying next to a tRNA gene	% of CAGs lying next to a tRNA gene
Thermococcales	*P. abyssi*	1993	95	4,77	24	25,26	3	32	1,61	48	6	12,50	80%	0.4	46	0	0	0	0
	P. horikoshii	2005	213	10,62	83	38,97	5	113	5,64	127	27	21,26	80%	0.5	46	1	2,17	2	40,00
	P. furiosus	2228	302	13,55	179	59,27	11	234	10,50	91	27	29,67	50%	0.5	46	2	4,35	2	18,18
	T. kodakaraensis	2357	455	19,30	249	54,73	14	294	12,47	257	104	40,47	50%	0.5	45	4	8,89	6	42,86
Thermoplasmatales	*T. acidophilum*	1530	71	4,64	21	29,58	2	26	1,70	39	7	17,95	50%	0.5	45	0	0	0	0
	T. volcanium	1548	106	6,85	77	72,64	5	97	6,27	31	16	51,61	70%	0.5	45	2	4,44	3	60,00
	P. torridus	1581	158	9,99	59	37,34	5	74	4,68	65	16	24,62	50%	0.6	43	2	4,65	3	60,00
Methanococcales	*M. aeolicus*	1552	115	7,41	41	35,65	3	50	3,22	43	21	48,84	60%	0.6	37	0	0	0	0
	M. jannaschii	1772	241	13,60	74	30,71	6	88	4,97	115	31	26,96	50%	0.5	37	1	2,70	1	16,67
	M. vannielii	1752	102	5,82	61	59,80	4	76	4,34	19	7	36,84	90%	0.5	37	2	5,41	4	100,00
	M. maripaludis	1772	99	5,59	42	42,42	3	59	3,33	12	2	16,67	50%	0.5	38	1	2,63	2	66,67
	M. maripaludis_c5	1879	145	7,72	88	60,69	7	120	6,39	36	23	63,89	90%	0.5	36	0	0	0	0
	M. maripaludis_c6	1888	196	10,38	137	69,90	9	182	9,64	62	43	69,35	50%	0.4	36	1	2,78	1	11,11
	M. maripaludis_c7	1855	179	9,65	118	65,92	8	146	7,87	21	17	80,95	60%	0.5	36	2	5,56	3	37,50
Halobacteriales	*H. marismortui*	3189	576	18,06	206	35,76	16	267	8,37	368	80	21,74	80%	0.4	48	7	14,58	11	68,75
	H. walsbyi	2875	786	27,34	570	72,52	32	732	25,46	205	105	51,22	50%	0.6	44	4	9,09	4	12,50
	Halobacterium sp.	2127	122	5,74	58	47,54	6	80	3,76	180	23	12,78	80%	0.4	47	1	2,13	1	16,67
	N. pharaonis	2726	549	20,14	251	45,72	18	328	12,03	240	70	29,17	50%	0.6	46	7	15,22	9	50,00
Methanosarcinales	*M. acetivorans*	4721	749	15,87	250	33,38	19	319	6,76	461	120	26,03	70%	0.5	59	2	3,39	3	15,79
	M. barkeri	3811	276	7,24	38	13,77	4	50	1,31	117	12	10,26	50%	0.5	62	1	1,61	1	25,00
	M. mazei	3437	289	8,41	79	27,34	9	101	2,94	124	23	18,55	70%	0.5	57	0	0	0	0
Sulfolobales	*S. acidocaldarius*	2329	175	7,51	47	26,86	2	59	2,53	105	8	7,62	50%	0.6	49	2	4,08	3	150,00
	S. solfataricus	3029	475	15,68	316	66,53	28	404	13,34	95	25	26,32	70%	0.4	46	4	8,70	6	21,43
	S. tokodaii	2874	462	16,08	224	48,48	18	295	10,26	266	90	33,83	70%	0.5	46	4	8,70	5	27,78
Pyrobaculum	*P. aerophilum*	2706	492	18,18	286	58,13	26	363	13,41	169	82	48,52	50%	0.5	*47*	1	2,13	2	7,69
	P. arsenaticum	2407	214	8,89	98	45,79	9	130	5,40	60	19	31,67	60%	0.5	*41*	2	4,88	4	44,44
	P. calidifontis	2200	129	5,86	26	20,16	3	35	1,59	71	7	9,86	60%	0.6	*22*	0	0	0	0
	P. islandicum	2062	139	6,74	42	30,22	4	50	2,42	40	10	25,00	80%	0.5	*45*	0	0	0	0
Prochlorococcus	*P. marinus*	1930	439	22,75	284	64,69	16	369	19,12	73	48	65,75	50%	0.5	40	5	12,50	8	50,00
	P. marinus_med4	1760	73	4,15	27	36,99	2	33	1,88	9	3	33,33	50%	0.6	38	1	2,63	1	50,00
	P. marinus_mit9312	1855	101	5,44	40	39,60	5	51	2,75	24	8	33,33	90%	0.5	40	1	2,50	2	40,00
	P. marinus_mit9313	2328	308	13,23	168	54,55	11	217	9,32	41	11	26,83	50%	0.5	45	3	6,67	4	36,36
	P. marinus_natl2a	1938	207	10,68	132	63,77	12	180	9,29	4	0	0	90%	0.6	39	3	7,69	3	25,00
Synechococcus	*Synechococcus_cc9605*	2749	746	27,14	613	82,17	36	750	27,28	239	211	88,28	90%	0.5	46	12	26,09	21	58,33
	Synechococcus_cc9902	2355	457	19,41	317	69,37	20	401	17,03	38	22	57,89	80%	0.5	45	6	13,33	10	50,00
	Synechococcus_pcc6301	2580	314	12,17	123	39,17	9	150	5,81	2	0	0	80%	0.5	45	2	4,44	3	33,33
	Synechococcus_pcc7942	2664	399	14,98	158	39,60	12	194	7,28	65	10	15,38	70%	0.5	45	6	13,33	10	83,33
	Synechococcus_wh8102	2581	586	22,70	446	76,11	24	530	20,53	104	68	65,38	80%	0.4	44	8	18,18	11	45,83
Leptospira	*L. borgpetersenii_jb197*	3242	134	4,13	41	30,60	3	50	1,54	2	0	0	50%	0.5	37	0	0	0	0
	L. borgpetersenii_l550	3273	224	6,84	77	34,38	8	129	3,94	1	0	0	80%	0.5	37	0	0	0	0
	L. interrogans	4768	1532	32,13	1064	69,45	80	1413	29,64	449	226	50,33	50%	0.6	37	4	10,81	5	6,25
Chlamydia	*C. felis*	1046	48	4,59	0	0	0	0	0	25	0	0	90%	0.5	38	0	0	0	0
	C. pneumoniae	1122	51	4,55	8	15,69	1	11	0,98	1	0	0	90%	0.5	37	0	0	0	0
	C. pneumoniae_ar39	1167	159	13,62	105	66,04	9	138	11,83	1	1	100,00	50%	0.6	38	0	0	0	0
	C. pneumoniae_j138	1107	53	4,79	8	15,09	1	10	0,90	1	0	0	90%	0.5	37	0	0	0	0
	C. pneumoniae_tw183	1155	114	9,87	66	57,89	7	82	7,10	1	0	0	50%	0.5	37	0	0	0	0
	C. caviae	1053	68	6,46	24	35,29	3	27	2,56	28	6	21,43	90%	0.5	38	1	2,63	1	33,33
	C. abortus	1003	36	3,59	0	0	0	0	0	4	0	0	60%	0.6	39	0	0	0	0
Mycobacterium	*M. avium_104*	5313	683	12,86	270	39,53	21	366	6,89	224	89	39,73	100%	0.4	47	4	8,51	5	23,81
	M. avium_paratuberculosis	4398	386	8,78	159	41,19	14	218	4,96	34	26	76,47	100%	0.6	46	5	10,87	7	50,00
	M. bovis	4003	473	11,82	269	56,87	27	337	8,42	1	0	0,00	70%	0.5	45	7	15,56	9	33,33
	M. leprae	2770	1081	39,03	890	82,33	58	1153	41,62	72	52	72,22	50%	0.5	46	16	34,78	25	43,10
	Mycobacterium_kms	5551	1074	19,35	691	64,34	47	902	16,25	4	4	100,00	50%	0.6	48	10	20,83	17	36,17
	Mycobacterium_mcs	5473	763	13,94	389	50,98	28	500	9,14	3	0	0	60%	0.6	49	6	12,24	10	35,71
	M. tuberculosis	4048	547	13,51	324	59,23	29	401	9,91	1	0	0	50%	0.5	45	4	8,89	5	17,24
	M. tuberculosis_cdc1551	4293	742	17,28	461	62,13	39	587	13,67	5	3	60,00	100%	0.5	45	6	13,33	8	20,51
	M. ulcerans	4981	1068	21,44	700	65,54	67	942	18,91	36	21	58,33	100%	0.5	45	9	20,00	12	17,91
	M. smegmatis	6938	1373	19,79	597	43,48	50	805	11,60	405	114	28,15	100%	0.6	50	7	14,00	11	22,00

Lactobacillus	*L. johnsonii*	1918	303	15,80	162	53,47	11	196	10,22	46	24	52,17	60%	0.5	79	3	3,80	4	36,36
	L. acidophilus	1938	467	24,10	317	67,88	27	432	22,29	88	51	57,95	50%	0.6	61	2	3,28	3	11,11
	L. brevis	2314	612	26,45	376	61,44	29	498	21,52	187	85	45,45	60%	0.5	66	7	10,61	10	34,48
	L. casei	2909	838	28,81	528	63,01	32	692	23,79	329	176	53,50	80%	0.4	60	3	5,00	6	18,75
	L. delbrueckii	2217	772	34,82	664	86,01	36	854	38,52	28	21	75,00	80%	0.4	95	12	12,63	20	55,56
	L. delbrueckii_baa-365	2040	584	28,63	416	71,23	26	543	26,62	101	61	60,40	60%	0.5	99	7	7,07	10	38,46
	L. gasseri	1898	428	22,55	283	66,12	16	352	18,55	50	24	48,00	50%	0.5	79	8	10,13	15	93,75
	L. plantarum	3136	836	26,66	558	66,75	38	728	23,21	194	87	44,85	70%	0.4	70	11	15,71	17	44,74
	L. sakei	1963	397	20,22	227	57,18	20	296	15,08	133	55	41,35	80%	0.5	63	4	6,35	6	30,00
	L. salivarius	2106	522	24,79	360	68,97	19	438	20,80	181	120	66,30	90%	0.5	78	3	3,85	6	31,58
Streptococcus	*S. pyogenes*	1805	177	9,81	160	90,40	7	190	10,53	2	2	100,00	50%	0.5	61	0	0	0	0
	S. pyogenes_m3	1951	310	15,89	287	92,58	6	338	17,32	1	1	100,00	60%	0.5	68	1	1,47	1	16,67
	S. pyogenes_mgas10394	1971	293	14,87	244	83,28	10	274	13,90	2	2	100,00	50%	0.5	67	2	2,99	2	20,00
	S. pyogenes_mgas5005	1950	219	11,23	160	73,06	5	189	9,69	1	0	0	50%	0.5	67	1	1,49	1	20,00
	S. pyogenes_mgas6180	1977	264	13,35	215	81,44	9	242	12,24	1	0	0	80%	0.5	65	1	1,54	2	22,22
	S. pyogenes_ssi1	1928	312	16,18	291	93,27	6	338	17,53	1	0	0	80%	0.5	57	0	0	0	0
Bacillus	*B. anthracis*	5630	1293	22,97	911	70,46	70	1168	20,75	1	0	0	80%	0.5	95	1	1,05	2	2,86
	B. anthracis_ames0581	5635	1141	20,25	717	62,84	52	911	16,17	1	0	0	80%	0.5	95	1	1,05	2	3,85
	B. anthracis_sterne	5415	1705	31,49	1384	81,17	92	1850	34,16	1	0	0	80%	0.5	95	1	1,05	2	2,17
	B. cereus	5476	1217	22,22	848	69,68	53	1098	20,05	45	40	88,89	80%	0.5	108	4	3,70	7	13,21
	B. cereus_atcc10987	5772	2073	35,91	1766	85,19	117	2343	40,59	137	113	82,48	80%	0.5	98	3	3,06	6	5,13
	B. cereus_zk	5269	952	18,07	582	61,13	49	757	14,37	15	13	86,67	80%	0.5	96	2	2,08	3	6,12
	B. clausii	4204	814	19,36	490	60,20	32	623	14,82	163	78	47,85	80%	0.5	74	4	5,41	5	15,63
	B. halodurans	4171	767	18,39	349	45,50	29	469	11,24	273	70	25,64	90%	0.5	78	3	3,85	4	13,79
	B. licheniformis	4290	739	17,23	431	58,32	24	526	12,26	53	18	33,96	80%	0.6	72	3	4,17	4	16,67
	B. licheniformis_dsm13	4289	631	14,71	309	48,97	19	394	9,19	47	15	31,91	80%	0.5	70	3	4,29	4	21,05
	B. thuringiensis	5261	853	16,21	457	53,58	40	591	11,23	13	8	61,54	70%	0.5	105	1	0,95	2	5,00
Staphylococcus	*S. aureus_col*	2724	215	7,89	120	55,81	8	153	5,62	1	1	100,00	80%	0.6	54	0	0	0	0
	S. aureus_mrsa252	2845	308	10,83	236	76,62	9	296	10,40	10	6	60,00	50%	0.7	60	1	1,67	1	11,11
	S. aureus_mssa476	2723	231	8,48	152	65,80	9	191	7,01	2	1	50,00	50%	0.6	60	0	0	0	0
	S. aureus_mu50	2775	257	9,26	213	82,88	12	263	9,48	2	2	100,00	90%	0.5	61	2	3,28	2	16,67
	S. aureus_mw2	2712	231	8,52	167	72,29	9	209	7,71	2	2	100,00	50%	0.6	61	1	1,64	1	11,11
	S. aureus_n315	2669	152	5,70	101	66,45	10	134	5,02	1	1	100,00	50%	0.6	63	1	1,59	1	10,00
	S. aureus_nctc8325	2969	380	12,80	221	58,16	7	269	9,06	1	0	0	100%	0.5	61	0	0	0	0
	S. aureus_rf122	2665	261	9,79	178	68,20	8	215	8,07	21	12	57,14	50%	0.6	60	1	1,67	1	12,50
	S. aureus_usa300	2648	218	8,23	157	72,02	7	206	7,78	1	1	100,00	50%	0.6	54	1	1,85	1	14,29
	S. epidermidis	2495	282	11,30	151	53,55	10	191	7,66	60	31	51,67	50%	0.7	60	0	0	0	0
	S. epidermidis_rp62a	2635	355	13,47	206	58,03	8	250	9,49	150	88	58,67	80%	0.6	62	0	0	0	0
	S. haemolyticus	2753	455	16,53	310	68,13	18	385	13,98	151	77	50,99	50%	0.6	60	2	3,33	3	16,67
Rickettsia	*R. prowazekii*	886	16	1,81	0	0	0	0	0	7	0	0	50%	0.5	33	0	0	0	0
	R. bellii	1469	142	9,67	44	30,99	6	59	4,02	17	1	5,88	50%	0.5	35	1	2,86	2	33,33
	R. conorii	1414	226	15,98	123	54,42	12	157	11,10	8	3	37,50	50%	0.5	34	3	8,82	4	33,33
	R. felis	1439	181	12,58	83	45,86	7	103	7,16	22	7	31,82	70%	0.5	34	3	8,82	5	71,43
	R. typhi	919	688	74,86	456	66,28	0	0	0	22	0	0	70%	0.4	34	0	0	0	0
Helicobacter	*H. pylori_hpag1*	1576	69	4,38	9	13,04	1	11	0,70	13	0	0	70%	0.5	36	0	0	0	0
	H. pylori	1630	118	7,24	64	54,24	3	77	4,72	37	19	51,35	70%	0.5	36	1	2,78	1	33,33
	H. acinonychis	1654	224	13,54	133	59,38	12	156	9,43	58	24	41,38	50%	0.5	36	0	0	0	0
	H. hepaticus	1915	443	23,13	205	46,28	13	274	14,31	259	81	31,27	50%	0.4	37	0	0	0	0
Enterobacteria	*E. coli*	4411	504	11,43	348	69,05	18	398	9,02	3	3	100,00	70%	0.5	83	4	4,82	5	27,78
	E. coli_cft073	5589	1419	25,39	1088	76,67	33	1259	22,53	38	35	92,11	70%	0.6	89	12	13,48	19	57,58
	E. coli_o157	5453	1486	27,25	1284	86,41	38	1458	26,74	27	22	81,48	100%	0.5	98	24	24,49	26	68,42
	E. coli_o157j	5395	1405	26,04	1224	87,12	33	1367	25,34	20	19	95,00	90%	0.4	106	24	22,64	26	78,79
	S. typhi	4711	922	19,57	744	80,69	32	868	18,42	5	5	100,00	70%	0.6	79	11	13,92	15	46,88
	S. typhi_ty2	4645	805	17,33	630	78,26	31	746	16,06	1	1	100,00	100%	0.5	78	9	11,54	11	35,48
	S. typhimurium	4622	635	13,74	448	70,55	26	546	11,81	2	1	50,00	70%	0.5	86	7	8,14	10	38,46
	S. enterica_choleraesuis	4699	699	14,88	476	68,10	27	600	12,77	12	11	91,67	50%	0.5	85	6	7,06	8	29,63
	S. enterica_paratyphi	4403	646	14,67	456	70,59	27	550	12,49	1	0	0	90%	0.5	82	8	9,76	11	40,74
	Y. pestis	4113	766	18,62	502	65,54	34	619	15,05	1	1	100,00	100%	0.4	70	7	10,00	9	26,47
	Y. pestis_kim	4240	974	22,97	682	70,02	45	855	20,17	1	0	0	70%	0.6	74	9	12,16	10	22,22
	Y. pestis_mediaevails	4138	848	20,49	582	68,63	40	723	17,47	1	0	0	100%	0.5	72	8	11,11	9	22,50
	Y. pseudotuberculosis	4095	818	19,98	638	78,00	32	773	18,88	13	12	92,31	100%	0.5	85	4	4,71	6	18,75
	**Total**	351111,00					2377,00	47441,00		8428,00	3475,00	5121,15			6739	420		595	
