# Supplementary material for: A hidden reservoir of integrative elements is the major source of recently acquired foreign genes and ORFans in archaeal and bacterial genomes
Source: Genome Biol. 2009 Jun 16;10(6):R65. doi: 10.1186/gb-2009-10-6-r65 (PMC2718499; doi:10.1186/gb-2009-10-6-r65)
Supplement: Additional data file 2 — Figure S1 shows the statistical results of the sizes of CAGs' ORFs with respect to the size of core genes. Figure S2 shows the statistics of ORF and ORFan sizes. Figure S3 shows the results of a χ2 test on the frequencies of ORFans inside and outside CAGs. [file gb-2009-10-6-r65-S2.pdf]

Figure S1: One-way ANOVA analysis of sizes of ORF in CAGs and core genes.

(a) ORF sizes from different categories of CAGs and core genes. The meaning of letters are as follows: A, sizes of ORFs included in CAGs having between 4-10 ORFs; B, sizes of ORFs included in CAGs having between 11-20 ORFs; C, sizes of ORFs included in CAGs having between 21-30 ORFs; D, sizes of ORFs included in CAGs having between 31-40 ORFs; E, sizes of ORFs included in CAGs having between 41-50 ORFs; F, sizes of ORFs included in CAGs having more than 50 ORFs; Z, core genes sizes. (b) Data had a normal distribution and a one-way ANOVA was applied. The result shows that no significant differences between the analyzed groups.

Figure S2: One-way ANOVA analysis of gene sizes of ORFans against ORFs from different categories.

(a) ORFs and ORFans sizes. (b) Data had a normal distribution and a one-way ANOVA was applied. The result is significant and means that at least one analyzed group is different from the others. (c) TukeyHSD test confirms that ORFans are smaller than ORFs. Significant difference between groups are denoted by asterisks: '***' if p-value is below 0.001, '**' if p-value is below 0.01, '*' if p-value is below 0.05.

Figure S3: ORFans in CAGs.

(a) expected (blue) and observed (red) average frequencies of ORFans included in CAGs. ORFans are overrepresented in CAGs. (b) Details of the Chi-squared analysis made with the expected and observed frequencies of the 122 analyzed genomes. It confirms that ORFans are more abundant in CAGs than that expected by chance only.

(a)

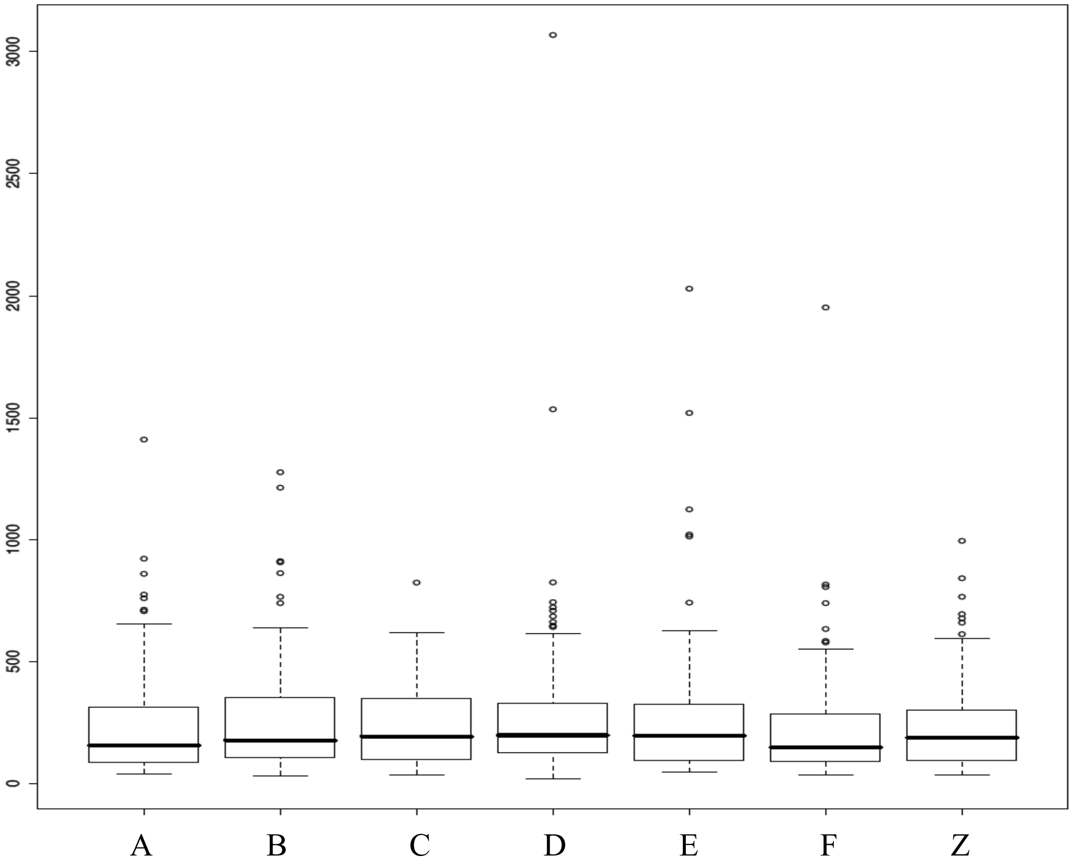

(b)

one-way ANOVA

	Df	Sum Sq	Mean Sq	F value	Pr(>F)
	**6**	**384147**	**64024**	**0.9773**	**0.4395**
Residuals	686	44938694	65508		

Figure S1

(a)

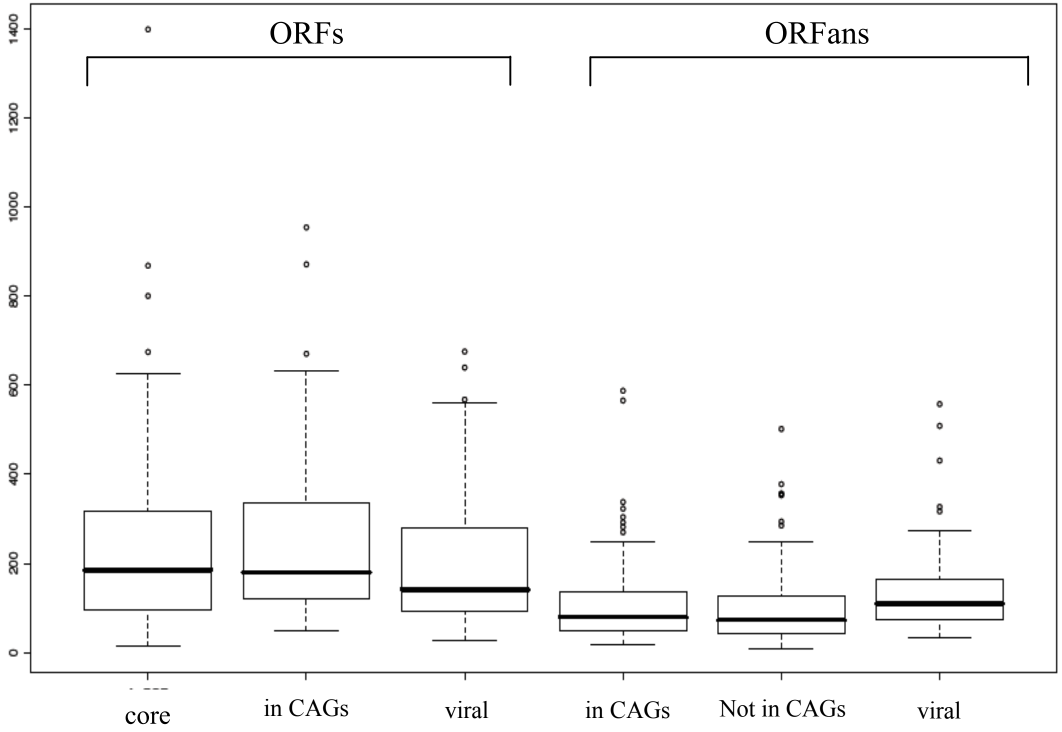

(b)

	Df	Sum Sq	Mean Sq	F value	Pr(>F)	
	**5**	**1771792**	**354358**	**17.707**	**2.308e-16**	***
Residuals	588	11767395	20013			

(c)

Tukey multiple comparisons of means
95% family-wise confidence level

Fit: aov(formula = V2 ~ V1, data = ORF-ORFans-sizes)

V1	V2	diff	lwr	upr	p	
**ORFs in CAGs**	**Core**	6.616162	-50.87282	64.105140	0.9994883	
**ORFs in viruses**	**Core**	-36.909091	-94.39807	20.579887	0.4435674	
**ORFans in CAGs**	**Core**	-117.080808	-174.56979	-59.591830	0.0000001	***
**ORFans not in CAGs**	**Core**	-128.404040	-185.89302	-70.915062	0.0000000	***
**ORFans in viruses**	**Core**	-97.666667	-155.15564	-40.177689	0.0000225	***
**ORFs in viruses**	**ORFs in CAGs**	-43.525253	-101.01423	13.963726	0.2560365	
**ORFans in CAGs**	**ORFs in CAGs**	-123.696970	-181.18595	-66.207992	0.0000000	***
**ORFans not in CAGs**	**ORFs in CAGs**	-135.020202	-192.50918	-77.531224	0.0000000	***
**ORFans in viruses**	**ORFs in CAGs**	-104.282828	-161.77181	-46.793850	0.0000044	***
**ORFans in CAGs**	**ORFs in viruses**	-80.171717	-137.66070	-22.682739	0.0010575	***
**ORFans not in CAGs**	**ORFs in viruses**	-91.494949	-148.98393	-34.005971	0.0000949	***
**ORFans in viruses**	**ORFs in viruses**	-60.757576	-118.24655	-3.268598	0.0312893	**
**ORFans nont in CAGs**	**ORFans in CAGs**	-27.707071	-61.66563943	6.251498	0.1342823	
**ORFans in viruses**	**ORFans in CAGs**	6.323232	-27.63533640	40.281801	0.8994311	
**ORFans in viruses**	**ORFans not in CAGs**	34.030303	0.07173431	67.988872	0.0493830	*

Figure S2

(a)

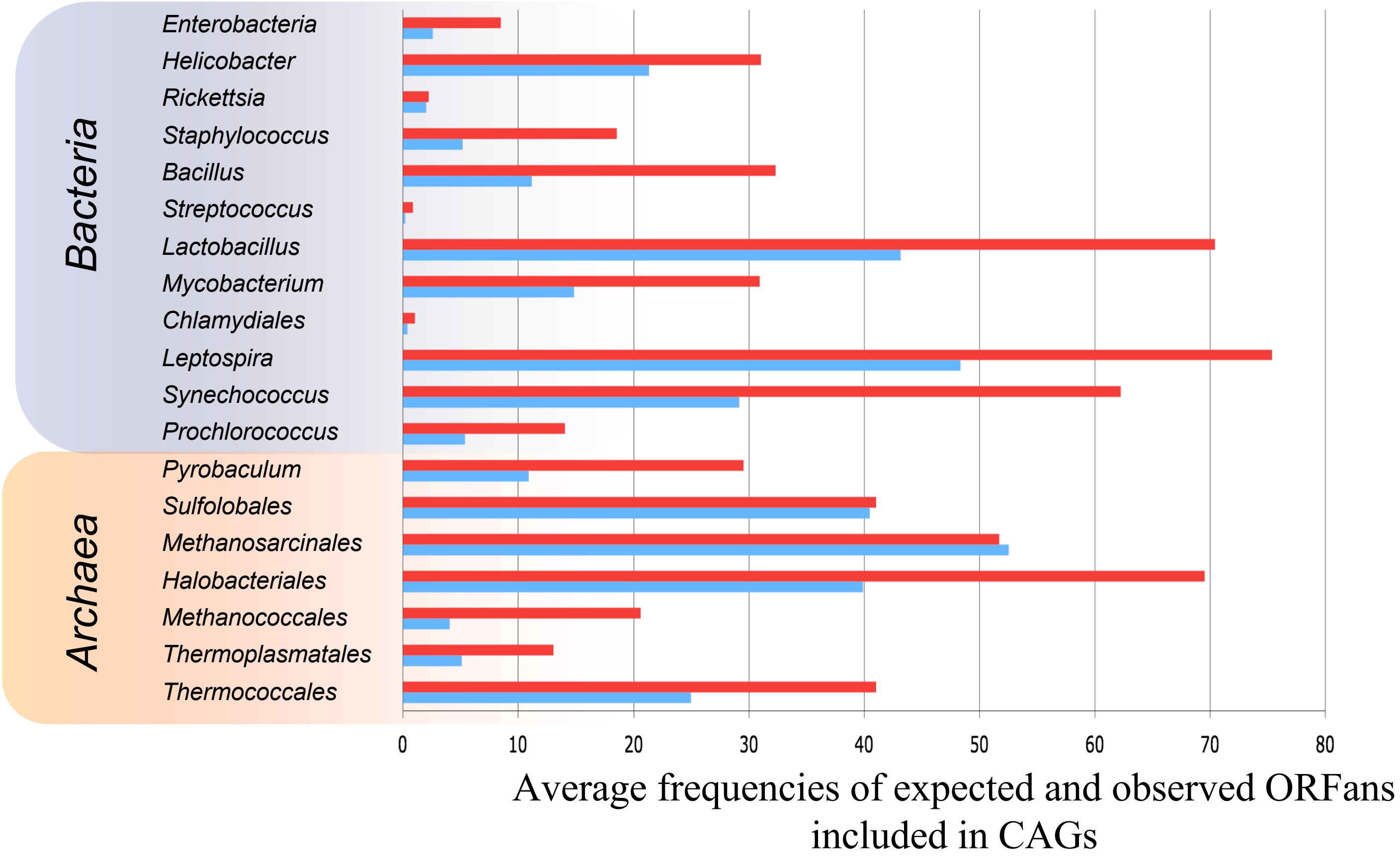

(b)

Chi-squared test for given probabilities

X-squared =1397.102, df = 102, p-value < 2.2e-16 ***

Figure S3
